# Excitotoxicity, calcium and mitochondria: a triad in synaptic neurodegeneration

**DOI:** 10.1186/s40035-021-00278-7

**Published:** 2022-01-25

**Authors:** Manish Verma, Britney N. Lizama, Charleen T. Chu

**Affiliations:** 1 Department of Pathology, University of Pittsburgh School of Medicine, Pittsburgh, PA 15261 USA; 2 Department of Ophthalmology, University of Pittsburgh School of Medicine, Pittsburgh, PA 15261 USA; 3 Pittsburgh Institute for Neurodegenerative Diseases, University of Pittsburgh School of Medicine, Pittsburgh, PA 15261 USA; 4 McGowan Institute for Regenerative Medicine, University of Pittsburgh School of Medicine, Pittsburgh, PA 15261 USA; 5 Center for Protein Conformational Diseases, University of Pittsburgh, Pittsburgh, PA 15261 USA; 6 Center for Neuroscience, University of Pittsburgh, Pittsburgh, PA 15261 USA; 7 Present Address: Astellas Pharma Inc., 9 Technology Drive, Westborough, MA 01581 USA

**Keywords:** Mitochondrial calcium, Mitochondrial calcium uniporter, NCLX antiporter, Parkinson’s disease, Alzheimer’s disease, LRRK2, PINK1, Beta-amyloid, Mitophagy, Excitotoxicity, Amyotrophic lateral sclerosis, Huntington’s disease, Glucocerebrosidase

## Abstract

Glutamate is the most commonly engaged neurotransmitter in the mammalian central nervous system, acting to mediate excitatory neurotransmission. However, high levels of glutamatergic input elicit excitotoxicity, contributing to neuronal cell death following acute brain injuries such as stroke and trauma. While excitotoxic cell death has also been implicated in some neurodegenerative disease models, the role of acute apoptotic cell death remains controversial in the setting of chronic neurodegeneration. Nevertheless, it is clear that excitatory synaptic dysregulation contributes to neurodegeneration, as evidenced by protective effects of partial *N*-methyl-*D*-aspartate receptor antagonists. Here, we review evidence for sublethal excitatory injuries in relation to neurodegeneration associated with Parkinson’s disease, Alzheimer’s disease, amyotrophic lateral sclerosis and Huntington’s disease. In contrast to classic excitotoxicity, emerging evidence implicates dysregulation of mitochondrial calcium handling in excitatory post-synaptic neurodegeneration. We discuss mechanisms that regulate mitochondrial calcium uptake and release, the impact of LRRK2, PINK1, Parkin, beta-amyloid and glucocerebrosidase on mitochondrial calcium transporters, and the role of autophagic mitochondrial loss in axodendritic shrinkage. Finally, we discuss strategies for normalizing the flux of calcium into and out of the mitochondrial matrix, thereby preventing mitochondrial calcium toxicity and excitotoxic dendritic loss. While the mechanisms that underlie increased uptake or decreased release of mitochondrial calcium vary in different model systems, a common set of strategies to normalize mitochondrial calcium flux can prevent excitatory mitochondrial toxicity and may be neuroprotective in multiple disease contexts.

## Background

Glutamate is the most commonly engaged neurotransmitter in the mammalian central nervous system, acting to mediate excitatory neurotransmission. However, excessive glutamatergic input elicits excitotoxicity, contributing to neuronal cell death following acute hypoxic-ischemic insults [1] or traumatic brain injury [2]. Moreover, sublethal glutamatergic injury contributes to dendritic degeneration [3] accompanied by mitochondrial calcium toxicity [4].

Glutamate binds to calcium-permeable ionotropic receptors called *N*-methyl-*D*-aspartate (NMDA) receptors (NMDARs) and α-amino-3-hydroxy-5-methyl-4-isoxazole propionic acid (AMPA) receptors (AMPARs) due to their ability to be activated by NMDA or AMPA, respectively. NMDAR and AMPAR are localized predominantly to dendritic spines, but also exist in perisynaptic and extrasynaptic regions [5]. Glutamate signaling is terminated to some extent by re-uptake into presynaptic terminals of neurons; however, glial cells play a predominant role in scavenging free glutamate via high-affinity glutamate transporters. Glutamate is converted into glutamine within glial cells, before being released for neuronal uptake as a starting material to replenish both excitatory (glutamate) and inhibitory (GABA) neurotransmitters (reviewed in [6]).

In classic excitotoxicity, ischemic injury results in ATP depletion and impaired glutamate transporter function. The resulting elevation in extracellular glutamate elicits a massive influx of sodium and calcium into neurons via NMDARs [7]. Sodium influx results in swelling of neurons, which is often reversible, whereas elevated calcium contributes ultimately to irreversible excitotoxic injury (reviewed in [8]). In addition to calpains and other degradative enzymes, death associated protein kinase 1 (DAPK1) and neuronal nitric oxide synthase (nNOS), which are associated with the NMDAR cytosolic tail, are activated [9, 10], while CREB signaling is downregulated [11]. Following the initial glutamate-stimulated calcium influx, other mechanisms that elevate cytosolic calcium are triggered, contributing to neuronal cell death [12]. Interestingly, synaptic NMDAR-mediated calcium flux elicits greater neurotoxicity when compared to other calcium sources [7], such as extrasynaptic NMDARs [13] or voltage-gated calcium channels [14]. A similar level of calcium elevation that is toxic after NMDAR activation may not be toxic when calcium enters via voltage-gated calcium channels. These observations suggest that the elevated calcium levels may interact with other factors regulated by the route of entry to determine toxicity.

The role of acute apoptotic [15–18] or non-apoptotic cell death [18–20] is less certain in chronic neurodegenerative diseases than in stroke and trauma. Indeed, loss of synaptic function and synaptic/dendritic atrophy likely occur well before the onset of cell death, along with interruptions in axonal transport [21], protein and organellar homeostasis [22], and chronic mitochondrial dysfunction [23, 24]. While excitotoxic cell death has also been reported in models of Parkinson’s disease (PD) [25], 1-methyl-4-phenyl-1,2,3,6-tetrahydropyridine (MPTP) intoxication is relatively acute, mediated by inhibition of mitochondrial complex I activity. Nevertheless, it is clear that excitatory synaptic dysregulation contributes to neurodegeneration, as evidenced by the protective effects of partial NMDAR antagonists [3, 26–28]. In general, synaptic activity is regulated by a balance of excitatory and inhibitory inputs. Synaptic hyperactivity may result from changes that favor excitatory inputs and/or increased responsiveness to a given stimulus, or from inhibitory deficits. While physiological bursts in activity trigger dendritic remodeling that underlies plasticity, chronically elevated synaptic activity may trigger pathological effects. In particular, an emerging body of literature implicates a role for mitochondrial calcium dysregulation downstream of synaptic hyperactivity.

**Fig. 1:**
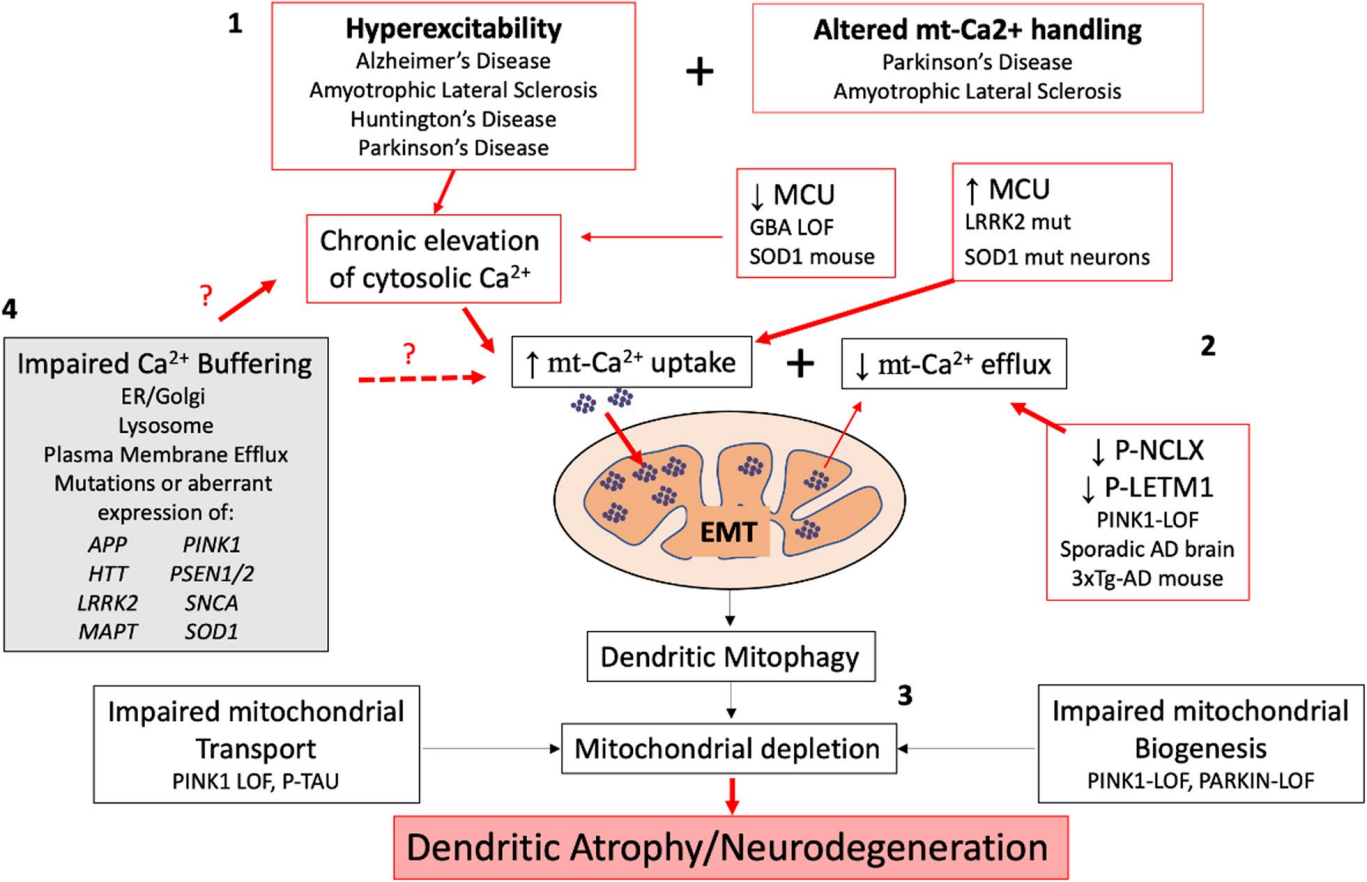
A combination of factors conspire to promote excitatory mitochondrial toxicity (EMT). (1) Pathophysiological processes linked to neuronal hyperexcitability have been identified in several neurodegenerative disorders. Chronic elevations in cytosolic calcium flux due to neuronal hyperexcitability elicit increased demand for mitochondrial calcium buffering. When hyperexcitability is combined with altered mitochondrial calcium handling and/or other factors that enhance susceptibility to mitochondrial injury, EMT may occur. (2) A set of intrinsic changes in mitochondrial calcium handling are triggered by familial neurodegeneration gene mutations that elicit increased mitochondrial calcium uptake and/or decreased mitochondrial calcium release. Mutations in *LRRK2* and *SOD1* increase MCU expression, while *PINK1* loss-of-function results in decreased phosphorylation and activities of NCLX and LETM1. NCLX is also decreasd in sporadic AD brains and tau inhibits NCLX activity. In some contexts, MCU expression is decreased rather than increased. This has been proposed as a compensatory response, but may still contribute to EMT by exacerbating cytosolic calcium elevations. Alternatively, MCU may be regulated differently in neurons than in glia. (3) Post-synaptic mitochondria are most vulnerable to EMT, and injured mitochondria are removed from dendrites by mitophagy. Elevated mitophagy that is not balanced by mitochondrial replacement results in mitochondrial depletion from dendrites. LOF mutations in the genes encoding PINK1 and Parkin impair mitochondrial biogenesis, while PINK1 mutations and hyperphosphorylation of tau contribute to impaired mitochondrial transport. The resulting loss of mitochondrial support leads to dendritic atrophy, an early sign of neurodegeneration. (4) Many neurodegenerative disease-associated genes also elicit altered calcium handling in other organelles. These alterations, particularly those at the ER-mitochondrial contact sites, may also contribute to dysregulation of cytosolic and/or mitochondrial calcium, although the exact relationships have not been directly addressed. Neurodegenerative disease-linked proteins (genes) include amyloid precursor protein (*APP*), glucocerebrosidase (*GBA*), huntingtin (*HTT*), leucine-rich repeat kinase 2 (*LRRK2*), Tau (*MAPT*), PTEN-induced kinase 1 (*PINK*), presenilin 1 and 2 (*PSEN1/2*), alpha-synuclein (*SNCA*) and superoxide dismutase 1 (*SOD1*). Mut, mutation; LOF, loss-of-function

In the following sections, we will discuss the regulation of cytosolic and mitochondrial calcium in neurons. We will review the evidence for sublethal excitatory injuries in models of familial neurodegeneration associated with PD, Alzheimer’s disease (AD), amyotrophic lateral sclerosis (ALS) and Huntington’s disease (HD), which highlight the key role of mitochondrial calcium dysregulation in excitatory post-synaptic injury that leads to dendritic degeneration. While the mechanisms underlying the increased mitochondrial calcium uptake or decreased release vary among the different model systems (Fig. 1), a common set of strategies to normalize mitochondrial calcium flux appears to be neuroprotective in several disease contexts.

## Calcium regulation and mitochondria in neuronal sub-compartments

Calcium plays an important role in multiple signaling cascades within neurons. It is essential for both pre-synaptic and post-synaptic processes, as well as vesicular transport, cytoplasmic motility and cell death, which require exquisitely precise, spatially separated waves of calcium increase and decrease. Dendritic spines serve to isolate and concentrate calcium signals arising from synaptic activity [29]. Mitochondria contribute to rapid, post-stimulatory calcium recovery by taking up massive amounts of calcium [30] and then releasing calcium more gradually back into the cytosol. Other factors that are important for calcium signal recovery include NMDAR/AMPAR channel inactivation, calcium sequestration into the endoplasmic reticulum, and extrusion of calcium from neurons via plasma membrane sodium-calcium exchangers. Mitochondrial function is also important for powering ATP-dependent calcium pumps [31].

Healthy mitochondria in the perisynaptic region, particularly in excitatory neurons, act to transiently take up calcium and slowly release it back to the cytoplasm [32]. Cytosolic calcium concentration is usually 50–100 nM at baseline. When high-calcium microdomains are generated near mitochondria, as may be observed when the overall level of calcium exceeds 500 nM, mitochondrial calcium uptake is triggered [33]. Free calcium within the mitochondrial matrix is buffered by binding to proteins and chemicals to form insoluble calcium phosphate and calcium carbonate precipitates. This allows mitochondria to hold higher levels of calcium for longer periods while continuing to respond to transient elevations of cytosolic calcium. Once the free mitochondrial calcium rises to a threshold, it will be released back to the cytosol through efflux mechanisms. Thus, mitochondria have an immense capability to fine-tune cytosolic and mitochondrial calcium fluxes in a localized fashion within neurons.

The major protein complex involved in mitochondrial calcium uptake is the mitochondrial calcium uniporter (MCU) [34, 35]. This is a high-capacity, low-affinity uptake system, ensuring that the rapid, high-capacity calcium uptake into mitochondria is not triggered until the mitochondria experience a spike of high calcium levels in the vicinity, such as during synaptic transmission [36]. The thresholds and levels of MCU function are regulated by accessory proteins, including mitochondrial calcium uptake 1 (MICU1) [37], MICU2 [38], essential MCU regulator (EMRE) [39], mitochondrial calcium uniporter regulator 1 (MCUR1) [40] and MCUb [41]. Interestingly, the overall affinity of the MCU complex to calcium is higher in neurons compared to other cell types, due to the expression of a brain specific MICU3 isoform [42]. This allows axonal mitochondria to take up calcium in response to smaller changes in cytoplasmic calcium, thus facilitating calcium-dependent acceleration of local ATP synthesis.

Mitochondrial calcium uptake is balanced by the activity of proteins such as mitochondrial sodium calcium exchanger (NCLX) [43] and leucine zipper EF-hand containing transmembrane protein 1 (LETM1) [44], which release calcium back into the cytosol. NCLX is a sodium/calcium antiporter at the inner mitochondrial membrane that is important for mitochondrial calcium release in excitable cells such as neurons and muscle cells [45–47]. While LETM1 mediates calcium uptake in response to moderate increases in cytosolic calcium, it has also been proposed to show reversible activity allowing for calcium extrusion from the matrix [36]. The endoplasmic reticulum-mitochondrial contact sites also act to regulate mitochondrial calcium homeostasis [48].

## Role of mitochondrial calcium homeostasis in synaptic transmission

Calcium impacts various mitochondrial processes including the activation of several metabolic enzymes to enhance ATP generation, increasing buffering capacity to prevent neuronal toxicity, and modulating mitochondrial transport (anterograde/retrograde) within the cell [49–52]. During synaptic transmission, perisynaptic mitochondria can buffer changes in calcium levels while maintaining the ATP balance [53]. Physiological levels of calcium uptake into the mitochondrial matrix enhance respiratory function, promoting ATP generation near sites of synaptic activity [54–56]. Increased cytosolic calcium generated during synaptic activity also acts to halt mitochondrial trafficking, resulting in mitochondrial accumulation near active synapses [57–59]. Recruitment of mitochondria to presynaptic terminals results in homeostatically decreased neurotransmitter release in response to prolonged activity [60]. Dysregulation of mitochondrial buffering capacity in conditional Liver kinase B1 (LKB1)-knockout neurons, which have impaired MCU activity [61, 62], leads to neuron hyperexcitability. LKB1 conditional knockout neurons show reduced axonal branching and decreased immobilization of mitochondria at nascent presynaptic sites [63]. In neurons, dendritic spines undergo waves of calcium transients. While free intracellular cytosolic calcium levels remain relatively low, approximately 50–100 nM, the localized calcium levels within spines may reach 10 μM during activation [64]. Taken together, these studies indicate that the mitochondrial calcium buffering capacity acts to prevent neuronal hyperexcitability [61].

In contrast to transient physiological calcium fluxes, sustained high levels of cytosolic calcium may lead to mitochondrial injury from calcium overload [65]. During classic excitotoxicity, excess mitochondrial calcium uptake can elicit reactive oxygen species (ROS) production [66], permeability transition pore opening [67], and cell death [68]. Further promoting mitochondrial calcium uptake via MCU overexpression exacerbates the NMDAR-mediated mitochondrial depolarization and excitotoxic cell death [69]. NADPH oxidase has been reported as a source of ROS following NMDAR activation [70]. Indeed, cell death caused by NMDA/AMPA/kainate receptor activation is attributed to ROS generation [71]. In the absence of oxygen, the agonist-induced cell death is attenuated, although cells are still capable of increasing intracellular calcium levels. This suggests that ROS and reactive nitrogen species (RNS) may be important down-stream effectors of toxicity elicited by increased intracellular calcium. Interestingly, nNOS-expressing neurons are resistant to the increase in NO mediated by NMDAR activation, possibly because of the increased expression of the antioxidant Mn superoxide dismutase in these cells [72]. Finally, protein–protein interactions involving NMDAR may also modulate the excitotoxic responses. The interaction of postsynaptic density-95 (PSD95) with NMDAR enhances the synthesis of NO, and suppression of PSD95 expression reverses the effects on NO without affecting cytosolic calcium uptake [73]. Similary, inhibiting the interaction of PSD95 with NMDAR using peptides prevents the ischemic brain damage induced by excitotoxicity [74]. Therefore, while elevated intracellular calcium is necessary for excitotoxic injury, it is not the only factor that must be considered.

## Mitochondrial calcium dysregulation in neurodegenerative diseases

While the role of acute excitotoxic cell death remains unclear under chronic neurodegenerative conditions, recent studies have indicated a pathogenic role for excitatory mitochondrial calcium dysregulation in mediating sublethal dendritic atrophy observed in chronic neurodegenerative diseases. Simplification and atrophy of dendritic structures are observed in post-mortem studies of AD, PD, and ALS [75–78] and their animal models [79–82]. Inhibiting calcium uptake is frequently reported to be neuroprotective [3, 83–85], and MCU inhibitors are neuroprotective in multiple genetic models of chronic neurodegenerative diseases [4, 86–89].

These studies implicate mitochondrial calcium dysregulation, rather than inappropriate activation of cytosolic calcium-dependent enzymes, as the major mechanism by which increased excitatory neurotransmission triggers mitochondrial depletion from and retraction of dendritic structures. Below, we review the evidence that excitatory mitochondrial toxicity (EMT) mediates the phenotype of excitotoxic dendritic degeneration observed in several familial neurodegenerative disease models.

### LRRK2 and excitatory mitochondrial toxicity

*LRRK2*, which encodes leucine-rich repeat kinase 2, is mutated in autosomal dominant PD. Dendritic atrophy is a prominent feature of neurons expressing disease-linked mutations in *LRRK2* [3, 4, 80, 84, 90–92], in which alterations of microtubule dynamics, endolysosomal dynamics and/or autophagy have been implicated (reviewed in [93]).

Electrophysiological studies indicate that mutant LRRK2 elicits hyperexcitability in cortical and hippocampal neurons through postsynaptic mechanisms [3, 94]. Presynaptic mechanisms have also been proposed given that LRRK2 is identified in synaptic vesicle fractions [95], and overexpression of LRRK2 G2019S decreases endocytosis and increases exocytosis [96]. A major group of LRRK2 targets involves a family of proteins called Rabs (reviewed by [97]), which are involved in membrane trafficking and recycling of synaptic vesicles [98, 99]. Proteomic analysis of *Drosophila* neurons expressing LRRK2-R1441C has identified differentially expressed or phosphorylated proteins involved in synaptic vesicle transmission [100]. LRRK2 has also been shown to regulate the voltage-gated calcium channels (CaV2.1), which are responsible for the influx of calcium ions in response to membrane depolarization [101].

The process of EMT as a mediator of excitatory dendritic injury was first described in mutant *LRRK2* models. Primary cortical neurons transfected with either LRRK2-G2019S or LRRK2-R1441C exhibit increased activity-dependent calcium influx through glutamate receptors and L-type calcium channels [3, 84]. Decreased density of mitochondria is observed specifically in post-synaptic structures, which precedes dendritic retraction [84]. The dendritic shrinkage can be blocked by inhibiting autophagy [84] or expressing an inactivating phosphomimic of the autophagy protein LC3 (microtubule-associated protein 1 light chain 3) at S/T12 [102], a site near the binding motif for cardiolipin-mediated mitophagy [103]. The use of genetically encoded calcium sensors established that both LRRK2-G2019S and LRRK2-R1441C elicited increased calcium uptake into mitochondria following neuronal depolarization [4]. The increased dendritic mitochondrial calcium uptake was mediated by transcriptional upregulation of MCU and MICU1 in mutant LRRK2 patient fibroblasts, and persisted even in permeabilized neurons exposed to identical “cytosolic” calcium concentrations. There were no changes in the expression of MICU2, which elevates the threshold for MCU activation, or NCLX, which extrudes calcium from mitochondria [4]. Neurons treated with MCU inhibitors, MCU RNAi or constitutively active forms of NCLX were protected from dendritic mitophagy and dendritic atrophy [4]. These data implicate calcium-dependent injury to dendritic mitochondria and their elimination by autophagy, mechanisms that link neuronal hyperexcitability to dendritic simplification.

### PINK1, Parkin and excitatory mitochondrial toxicity

PTEN (Phosphatase and tensin homolog)-induced kinase 1 (PINK1) and Parkin are products of two recessive PD genes that have been heavily implicated in regulating one of the pathways of selective mitophagy (reviewed in [104]). Both enzymes also have other functions, some of which impact calcium handling in neurons.

Like primary neurons expressing mutant *LRRK2*, *Pink1* knockout mice exhibit reduced dendritic arbors in midbrain, cortical and hippocampal neurons [81, 105, 106]. Older, but not young (6-month vs 2-month) *Pink1* knockout mice exhibit increased excitatory neurotransmission, accompanied by increased neurotransmitter release on KCl stimulation without changes in protein expression of synaptic vesicle proteins syntaxin1a, Munc-18 or SNAP25 [107]. Moreover, Pink1 is able to modulate sensory dendrite pathology in a *Drosophila* model of chemotherapy-induced neuropathy [108]. Neurochemical analysis in *Pink1* knockout rats showed higher glutamine levels as an indirect measure of glutamate neurotransmission [109]. Moreover, knocking down *Pink1* expression increases the density of thin spines and decreases the expression of postsynaptic cluster proteins, PSD95 and Shank. These changes are proposed to increase the susceptibility of *Pink1*-deficient neurons to excitotoxicity [106]. The reciprocal effect on PSD95 has also been observed, whereby overexpression of *PINK1* increases the expression of PSD95 [81]. *Pink1* knockout rat models also display increased glutamate release in striatal spiny neurons, accompanied by enhanced frequency and amplitude of spontaneous excitatory postsynaptic currents and increased numbers of synapses as measured by electron microscopy [110].

One of the earliest shRNA studies of PINK1 in SH-SY5Y neuroblastoma cells revealed mitochondrial calcium overload due to deficient function of the Na^+^/Ca^2+^ calcium exchanger, lowering the threshold for opening of the mitochondrial permeability transition pore [111]. Additional observations supporting a role for mitochondrial calcium overload derives from observations that inhibiting mitochondrial calcium uptake using ruthenium red restores Δ*Ψ*_m_ in cells co-expressing α-syn A53T and Pink1 W437X and rescues neurite outgrowth [112]. Moreover, mitochondria exhibit altered ultrastructure in PINK1-deficient cells [113]. Subsequently, PINK1 was found to facilitate NCLX activation through phosphorylation at a novel site by protein kinase A (PKA) [45]. PINK1 is able to directly phosphorylate the catalytic subunit of PKA [105], and either PKA or the NCLX phosphomimic is able to rescue the effects of PINK1 deficiency in PINK1 knockout primary neurons [45]. Subsequently, it was found that LETM1, another mitochondrial calcium transporter involved in the bidirectional movement of calcium [44], is a direct phosphorylation target of PINK1 [114].

Like neurons expressing mutant LRRK2 or deficient PINK1, loss of Parkin causes increased excitatory neurotransmission, but via different mechanisms. Parkin is an E3 ubiquitin ligase, which has been shown to be important for the pruning and degradation of excitatory synapses [115]. Loss of endogenous Parkin or expression of the PD-linked Parkin mutants increases the vulnerability to synaptic excitotoxicity. Parkin is also necessary for the maintenance of postsynaptic endocytosis of AMPARs [116], and acts to regulate the endoplasmic reticulum-mitochondrial crosstalk, with indirect effects on mitochondrial calcium levels [117]. However, whether or not these mechanisms contribute to EMT in Parkin-deficient models has yet to be studied.

### Beta-amyloid (Aβ), tau and excitatory mitochondrial toxicity

Calcium mishandling is also believed to contribute to the pathogenesis of AD [118, 119]. Among the pathological hallmarks of AD is the accumulation of extracellular amyloid plaques and intracellular tangles of tau.

Oligomeric Aβ elicits elevated intracellular calcium levels accompanied by spine loss in vivo [120]. Accumulation of Aβ on synaptic mitochondria leads to mitochondrial calcium overload and loss of mitochondrial membrane potential [121–123]. In addition, overexpression of Aβ results in decreased pre-synaptic mitochondria, decreased synaptic vesicles and elevated synaptic fatigue in *Drosophila* neurons [124]. In the APP/PS1 transgenic mouse model of AD, secreted Aβ elevates the calcium concentrations in mitochondria in neurons, which can be ameliorated by treatment with an MCU inhibitor [125]. Interestingly, post-mortem AD brains exhibit downregulated expression of calcium influx genes, such as *MCU*, *MICU1* and *MICU2*, but upregulated expression of *NCLX*. The authors of this study noted that such gene expression changes could be due to a compensatory response to chronic mitochondrial calcium overload. Only mRNA was examined in this study and it was unknown what stage(s) of disease or brain region(s) were analyzed. In contrast, another study showed decreased NCLX protein in both the 3xTg-AD mouse model and the frontal cortex of sporadic AD patients [126].

AD mouse models also show evidence of excitatory hyperactivity accompanied by decreases in mitochondrial buffering capacity [127]. In mice co-expressing mutated *APP*_swe_ and mutated *PS1*_G384A_, spontaneous increases in hyperactive neurons were observed early in the hippocampus, prior to the formation of plaques [128]. Mutations in presenilins promote calcium leakage into the cytosol through endoplasmic reticulum calcium overload, accompanied by post-translational modification and enhanced recruitment of neuronal ryanodine receptors [118, 129, 130]. These mutations also exaggerate the inositol triphosphate-evoked calcium release, without affecting the cytosolic calcium buffering [131]. It has also been suggested that mutations in presenilins affect intracellular calcium levels [132], and that elevations in intracellular calcium play key roles in promoting tau pathology [133] through activation of microtubule affinity-regulating kinase, cyclin-dependent kinase 5 or AMP-activated kinase [134]. Interestingly, tau inhibits the activity of NCLX, leading to a decrease in mitochondrial calcium efflux and activation of apoptotic cell death [135]. Loss of NCLX function has been shown to accelerate pathology and memory decline in 3xTg-AD mice [126]. In contrast, in AD models expressing a mutant form of PS2, decreased rather than enhanced mitochondrial calcium uptake was linked to glutamate-induced excitotocity. This may be attributed to energy-deprivation with reduced capacity to handle stress (in this case a lower dose of glutamate) [136].

### Mutant SOD1 and excitatory mitochondrial toxicity

Dysfunctional mitochondria contribute to the pathogenesis of both sporadic and familial ALS (reviewed in [137, 138]). Multiple genetic mutations have been identified in patients with familial ALS, the most common of which are *SOD1* and *C9orf-72.* Mutations in the *SOD1* gene encoding the Cu^2+^, Zn^2+^-dependent superoxide dismutase are found in 20% of patients with familial ALS [139]. Mice overexpressing mutant SOD1-G93A exhib i t motor neuron degeneration with sympoms relevant to human disease progression. Interestingly, this model also exhibits early deficits in mitochondrial calcium homeostasis. *SOD1* G93A tran sgenic mice exhibit a significant decrease in mitochondrial calcium loading capacity in motor neurons, which precedes the disease onset [140]. Furthermore, in a study comparing disease progression between transgenic and non-transgenic animals, increased MCU expression was observed in embryonic *SOD1* mutant motor neurons, while MCU downregulation was observed in adult presymptomatic and symptomatic *SOD1* mutant animals. The authors suspect that this decrease of MCU is likely a compensatory response to early calcium dyshomeostasis [141]. However, as the number of mitochondria and the mitophagy were not directly studied, it is unknown whether the decrease in MCU expression could be explained by decreased mitochondrial content.

### Mutant Huntingtin (mHTT) and excitatory mitochondrial toxicity

Mitochondrial calcium dyshomeostasis is also thought to be one of the causes for striatal neurodegeneration in HD. mHTT contains an abnormal expansion of polyglutamine at the N-terminus of HTT, which is unable to fold and is cleaved to form short peptides with a high propensity to aggregate. Mitochondria from HD patients exhibit decreased buffering capacity when challenged with a bolus of calcium compared to healthy controls, with mitochondria from juvenile-onset cases markedly worse than those from adult-onset cases [142]. Rodent mHtt transgenic models also exhibit mitochondrial calcium buffering dysfunction [143], which, interestingly, precedes the onset of motor dysfunction [142].

However, experiments using isolated mitochondria are limited by the removal of cellular contexts, and other factors such as ROS/RNS may modulate cellular susceptibility to excitotoxic injury. Within intact cells, mitochondria exhibit high respiratory control, and in this context, moderate expression of mHtt does not significantly impair mitochondrial respiration in resting young neurons. Yet, when challenged with calcium by transiently activating NMDARs, mitochondria from striatal neurons expressing mHtt fail to reestablish calcium homeostasis compared to wildtype [144]. Interestingly, a subset of neurons are spared in HD. Studies by Canzoniero et al. demonstrated that these neurons express high levels of nNOS. While they show similar calcium uptake and NMDAR-evoked responses as other neurons, these neurons have decreased ROS generation in response to NMDAR activation compared to cultured neurons expressing lower levels of nNOS [145].

## Similarities and differences between excitotoxicity and EMT

In classic excitotoxicity, the cytosolic calcium overload results in enzyme activation and acute cell death [8]. This is not the situation observed in most genetic models of neurodegenerative diseases, in which cell death is often not a prominent feature. This may be related to the difference in the level of calcium disruption. Much greater fluxes are triggered throughout the neuron during classic excitotoxic injury due to ischemia-mediated failure of ATP-dependent pumps, compared to the effects of chronically elevated excitatory postsynaptic potential frequency observed in several genetic models of neurodegeneration.

As discussed above, excitatory injury may be either exacerbated by or ameliorated by inhibition of mitochondrial calcium uptake via MCU. This may be related to the presumed site of calcium toxicity. In classic excitotoxicity, high cytosolic levels of calcium result in enzyme activation, leading to cell death. For example, in Gaucher’s disease (*GBA*^−/−^), increased sensitivity to excitotoxic injury has been attributed to a decrease of mitochondrial calcium uptake due to the decreased MCU expression [146]. Notably, Gaucher’s disease involves infantile presentation of disease, whereas heterozygous *GBA* mutations have been implicated in PD. Insufficient mitochondrial calcium buffering would be predicted to contribute to increased sensitivity to classic cytosolic calcium toxicity mechanisms as opposed to excitatory mitochondrial toxicity. In this context, enhanced uptake of calcium by mitochondria, endoplasmic reticulum and other organelles acts to reduce injury by reducing the cytosolic calcium level.

Yet elevated calcium uptake via MCU can also lead to cell death during acute injury. In stroke, there is a biphasic response to CsA treatment, suggesting an initial phase during which glutamate toxicity can be reversed, which is not dependent on the energy reserve of the cell. However, after prolonged exposure, there is a collapse in the ATP content of the cell. Under these circumstances, mitochondrial calcium uptake activates PARP (poly-adenosine ribosyl polymerase) to trigger NADH depletion and mitochondrial membrane potential collapse [147].

In EMT, it is the mitochondria themselves that are injured, triggering mitophagy, mitochondrial depletion from dendrites and dendritic atrophy (Fig. 1). The injured mitochondria undergo mitophagy, leading to insufficient mitochondrial support of dendritic structures. Inhibiting mitochondrial calcium uptake by MCU, or facilitating recovery by regulated efflux through NCLX, can protect dendritic mitochondria and dendritic structures.

While several genetic mutations discussed above result in hyperexcitability of synaptic function, which will cause localized increases in intracellular calcium, calcium levels alone do not determine toxicity. Instead, emerging evidence implicates multifactorial pathogenesis. As discussed above, the site of calcium entry and the presence or absence of antioxidant defenses against ROS/RNS may also regulate susceptibility to excitatory injury.

Likewise, while hyperexcitability is a driving factor for EMT, it is not the only factor. For instance, elevated synaptic activity may be combined with intrinsic dysregulation of mitochondrial calcium transporters (Fig. 1). Permeabilization experiments have shown that the increased mitochondrial calcium uptake in the dendrites of mutant LRRK2- expressing neurons persists even after the elevations of cytosolic calcium caused by synaptic hyperexcitability have been controlled [4]. Moreover, a recent study demonstrated that MCU overexpression is sufficient to cause pathology in the absence of synaptic hyperactivity [148]. This may reflect changes in mitochondrial calcium transport protein expression, post-translational modification and/or function that are induced by genetic mutations.

Finally, the potential contributions of altered  endoplasmic reticulum, Golgi, lysosomal and plasma membrane calcium pump function remain to be studied in the context of EMT. Disruptions in calcium handling by lysosomes have been repo rted in models of PD and ALS [149, 150]. Certainly, any change that promotes even mild, chronic elevations in cytosolic calcium has the potential to enhance calcium uptake by mitochondria and/or to im pair their rec overy from an episode of calcium buffering.

## Neuroprotective strategies for EMT

Neuroprotection against EMT and excitatory dendritic degeneration could conceivably be achieved by targeting multiple processes. These include strategies to normalize the changes observed in the different model systems at the level of (1) presynaptic vesicle exocytosis, (2) post-synaptic calcium uptake, (3) enhanced mitochondrial quality, (4) preventing overactivation of mitochondrial calcium uptake, and (5) promoting the regulated release of calcium out of mitochondria.

As mechanisms underlying increased excitatory neurotransmission are fairly different from model to model, the first strategy of reducing excess glutamate release may be difficult to achieve. In contrast, numerous studies have shown the benefit of blocking calcium uptake through either glutamate receptors or *L*-type calcium channels [3, 83, 84, 151–153]. Yet, the impact of inhibiting these pathways in the more complex human in vivo setting is difficult to predict, particularly if the mechanism of injury involves intrinsic changes to mitochondrial calcium handling or mitochondrial dynamics (Fig. 1) that are not corrected by blocking extracellular calcium uptake. Clinical trials on most NMDAR antagonists have failed to demonstrate benefit for stroke and traumatic brain injury, with side effects triggering early termination [154]. Given the differences in mechanism that may be triggered during acute brain injury relative to chronic neurodegeneration, it is interesting to note that the low-affinity NMDAR antagonist memantine did show small, but significant benefits for moderate to severe AD [27] and has been approved for clinical use.

Mitochondrial quality control is mediated by localized degradation and repair, including the mitochondrial unfolded protein response, or by whole organelle turnover through autophagy [155]. Basal mitophagy is undoubtedly important for maintaining mitochondrial resilience. Impaired calcium recovery [156] and elevated basal mitophagy have been observed in neuronal cells [113, 155] and in pancreatic beta cells in vivo [157], evidently through one of the several PINK1- and Parkin-independent mechanisms [103, 158, 159]. Basal mitophagy in vivo also appears to be mediated by PINK1/Parkin-independent pathways [157, 160–162]. Induced mitophagy can be regulated by at least three distinct mechanisms: PINK1/Parkin-induced mitochondrial ubiquitination, externalization of the inner mitochondrial lipid cardiolipin, and transcriptional upregulation of mitophagy receptors such as FUNDC1 [104]. In settings where PINK1 and Parkin are not mutated, small molecules that serve to enhance endogenous PINK1 activity [163] or expression [164] may show beneficial effects.

However, the impact of augmenting injury-induced mitophagy is not straightforward to predict. While mitophagy generally promotes cell survival, a potential side effect of excessive mitophagy in neurons is the loss of synaptic structures due to mitochondrial depletion, particularly under disease conditions that impair mitochondrial biogenesis [165–167]. Dendritic degeneration in culture models of LRRK2- and PINK1-related neurodegeneration is preceded by the loss of dendritic mitochondria [81, 84]. Enhanced autophagy has been shown to contribute to excitotoxic lesions in neonatal rats [168]. Parkin-mediated mitophagy has also been implicated in glutamate excitotoxicity in culture systems [169]. These factors may explain the perhaps unexpected observation that inhibiting autophagy or mitophagy acts to preserve dendritic arbors [170, 171]. Furthermore, the potential effects of modulating autophagy or mitophagy on other cell types in the brain, such as glial and vascular cells, remain to be delineated.

Perhaps the most promising avenue for protecting against neuronal atrophy and synaptic loss due to EMT involves the normalization of mitochondrial calcium flux. Knockdown of MCU and application of MCU inhibitors are effective in multiple neurodegenerative disease models [4, 88, 89, 172]. This is the case even when the primary mechanism of EMT involves impaired NCLX activity [45, 126]. Conversely, overexpressing NCLX or its constitutively active phosphomimic NCLX-S258D, confers protection against neuronal atrophy and dendritic loss even when the primary mechanism involves hyperactivation of MCU [4, 173]. In the appropriate familial disease setting, it may also be possible to use LRRK2 kinase inhibitors [174] or small molecules that augment PINK1 expression [164] to reverse downstream changes in mitochondrial calcium flux.

The potential contributions of other brain cell types must also be considered. While global MCU knockout does not show protection against hypoxia ischemic injury [175], neuronal specific conditional knockdown or siRNA-mediated knockdown of MCU has been shown to be neuroprotective in this injury model [176]. Furthermore, there may be tissue-specific minor pathways through with calcium ions can enter the mitochondria. In MCU-KO mice, isolated mitochondria from nonsynaptic and synaptic regions show slowed calcium uptake, which is inhibited by Ru360. However, mitochondria isolated from liver, heart and skeletal muscle tissue show completely inhibited calcium uptake [177].

Although numerous studies have shown that inhibiting MCU may be neuroprotective, it is important to keep in mind that these strategies may exacerbate classic excitotoxic mechanisms. Several acute and some chronic disease contexts involve decreased MCU activity [136, 146], and impaired mitochondrial uptake to begin with. Thus, it would be important to continue to define which steps of calcium homeostasis are affected in a given familial or sporadic neurodegeneration context (Fig. 1) to delineate those settings involving EMT-driven pathogenic mechanisms.

## Conclusion

Neurons respond differently to acute *vs* chronic excitotoxic stimuli. During acute excitotoxicity stimuli, due to energy crisis, neurons undergo cell death. This is preceded by a rapid influx of calcium into the cells, which further exacerbates the situation. In contrast, chronic excitotoxicity is elicited by a sublethal increases in excitatory neurotransmission. There is a gradual loss of neuronal function and dendritic atrophy, eventually leading to neuron loss.

Recent studies have conclusively shown that in addition to cytosolic calcium, mitochondrial calcium also plays an important role in regulating synaptic activity. Apart from being an ATP generator, mitochondria are important for calcium buffering, particularly in excitatory neurons.

Shortening of dendrites and loss of dendritic arborization can occur independently of or preceding neuronal cell death. Dendrites are important for segregating calcium signals and dendritic mitochondria play a vital role in buffering calcium during synaptic activity. Localized increases in calcium levels at synapses halt mitochondrial transport, accumulating them near synapses. Synapses lacking mitochondria in their vicinity show increased hyperexcitability, with loss of homeostatic downregulating mechanisms. Hyperactive neurons are often accompanied by sustained higher cytosolic calcium levels leading to mitochondrial calcium overload, which in turn increases mitochondrial ROS production and mitochondrial damage, leading to enhanced mitochondrial removal by autophagy.

Neurodegenerative diseases that show loss of mitochondria are particularly susceptible to excitotoxicity. For example, neurons expressing *LRRK2* mutants (G2019S or R1441C) show decreased dendritic mitochondrial content as well as increased mitochondrial calcium overload which can be prevented by inhibiting MCU activity [4]. Similarly, loss of PINK1 function induces hyperexcitability as evidenced by an increase in glutamine [109]. Recent studies have implicated PINK1 in the regulation of mitochondrial calcium homeostasis by regulating mitochondrial NCLX function through phosphorylation [45]. Like PINK1, loss-of-function mutation in Parkin also increases the vulnerability of neurons to synaptic excitotoxicity [115].

The role of calcium in the pathogenesis of AD, ALS and HD is also beginning to emerge. Exposure to oligomeric Aβ causes spine loss which might be associated with mitochondrial calcium overload, and loss of functional mitochondria. Deletion of NCLX accelerates memory decline and AD-type pathology in mouse models [126]. Improper calcium handling has also been shown to play a role in tau-mediated pathology [135]. Considering all the evidence, dysregulation of mitochondrial calcium homeostasis makes neuronal cells susceptible to excitotoxic injury. From a therapeutic point of view, preservation of mitochondrial function by targeting the mitochondrial calcium homeostasis machinery provides an attractive target to develop the next generation of drugs that can prevent or slow these devastating neurodegenerative disorders.

## Data Availability

Not applicable.

## Abbreviations

Aβ: Amyloid beta
EMT: Excitatory mitochondrial toxicity
LRRK2: Leucine-rich repeat kinase 2
MCU: Mitochondrial calcium uniporter
NCLX: Mitochondrial sodium calcium exchanger
NMDA: *N*-Methyl-d-aspartic acid
NMDAR: *N*-Methyl-d-aspartic acid receptor
PINK1: PTEN induced kinase 1
AMPA: α-Amino-3-hydroxy-5-methyl-4-isoxazolepropionate
AMPAR: α-Amino-3-hydroxy-5-methyl-4-isoxazolepropionate receptor
GABA: γ-Aminobutyric acid
LETM1: Leucine zipper and EF-hand containing transmembrane protein 1
LKB1: Liver kinase B1
